# 阿瑞匹坦预防化疗诱导恶心呕吐的疗效分析

**DOI:** 10.3779/j.issn.1009-3419.2018.10.12

**Published:** 2018-10-20

**Authors:** 莎莎 关, 丽沙 张, 殿胜 钟, 晴 马, 凡路 孟, 宜 邵, 涛 于, 夏 刘

**Affiliations:** 300052 天津，天津医科大学总医院肿瘤科 Department of Medical Oncology, Tianjin Medical University General Hospital, Tianjin 300052, China

**Keywords:** 阿瑞匹坦, 癌症, 化疗诱导的恶心呕吐, 顺铂, Aprepitant, Cancer, CINV, Cisplatin

## Abstract

**背景与目的:**

化疗是治疗恶性肿瘤最重要的手段，然而化疗诱导的恶心呕吐对患者影响深远。近些年来，不断有新的止吐药物问世，如阿瑞匹坦。我们回顾性分析了2014年8月-2016年12月使用含高度致吐药顺铂化疗方案的肿瘤患者中，阿瑞匹坦联合托烷司琼、地塞米松预防化疗诱导呕吐（chemotherapy-induced nausea and vomiting, CINV）的效果。

**方法:**

在使用含高度致吐药顺铂化疗方案的肿瘤患者中，应用阿瑞匹坦联合托烷司琼、地塞米松的止吐方案，观察期分为整体观察期：化疗后0 h-120 h；急性呕吐观察期：化疗0 h-24 h；延迟性呕吐观察期：化疗后24 h-120 h。观察目标包括完全应答（complete response, CR）和完全预防（complete protection, CP）。

**结果:**

整体观察期的CR为86.02%，急性呕吐观察期和延迟性呕吐观察期分别为89.25%、87.1%；CP分别为46.22%、83.87%、45.16%。止吐效果与年龄分布关系具有显著性（*P*=0.008）。

**结论:**

阿瑞匹坦联合托烷司琼、地塞米松的方案可有效预防使用含高度致吐药顺铂化疗方案的肿瘤患者中CINV的发生，提高患者的生活质量和化疗的依从性。

## Abstract (en)

**Background and objective:**

Chemotherapy is the most important method for cancer treatment. However, chemotherapy induced nausea and vomiting (CINV) has a profound effect on patients. In recent years, there have been new antiemetic drugs, such as aprepitant. We review the curative effect of aprepitant with tropisetron and dexamethasone for prevention of nausea and vomiting in patients receiving Cisplatin chemotherapy.

**Methods:**

Observation is divided into three stages. Whole study phase (0-120 h after chemotherapy administration), acute phases (0-24 h), and delayed phase (24 h-120 h). The primary endpoints were complete response (CR) and complete prevention (CP) during the three different study phase.

**Results:**

In the whole study phase, 86.02% of patients achieved CR; in acute phases and delayed phases were 89.25%, 87.1%, respectively. CP were 46.22%, 83.87%, 45.16%, respectively. Anti-CINV effect was significantly associated with age distribution (*P*=0.008).

**Conclusion:**

Aprepitant with tropisetron and dexamethasone prevented effectively CNIV for patients receiving Cisplatin chemotherapy. This combination could improve the quality of life and the compliance of patient with chemotherapy.

在世界范围内，肿瘤已成为威胁人类健康的第一大杀手，中国恶性肿瘤发病率为235.23/10万，死亡率为148.81/10万^[1]^。其中，肺癌每年新增患者180多万，占所有恶性肿瘤的13%，死亡近160万，占恶性肿瘤死亡率的19.4%^[2]^，两者均占恶性肿瘤的第一位^[3]^。目前，肿瘤的治疗不断发展，越来越多的靶向药物应用到临床，但化疗仍然是治疗肿瘤的基石。有些化疗药物可以诱导恶心呕吐（chemotherapy induced nausea and vomiting, CINV），造成患者的耐受性及依从性降低，甚至还会诱发一系列并发症，增加患者对化疗的恐惧感。为了改善患者的CINV，不断有新的止吐药物问世，阿瑞匹坦是神经激肽-1（neurokinin-1, NK-1）受体阻滞剂，可以高选择性阻断NK-1受体并与其紧密相结合，从而抑制化疗药物引起的呕吐，特别是对于预防延迟期呕吐疗效显著，近年来应用于临床。我们回顾分析了我科使用含高度致吐药顺铂化疗方案的肿瘤患者中，阿瑞匹坦联合托烷司琼、地塞米松预防CINV的效果。

## 1 资料与方法

### 1.1 化疗方案

我们回顾了2014年8月-2016年12月肿瘤科93例恶性肿瘤患者使用阿瑞匹坦预防顺铂引起的恶心呕吐的疗效，其中肺癌占69例（74.19%）；消化道肿瘤占24例（25.81%）。顺铂用量为（75 mg/m^2^-80 mg/m^2^），分为第1、2、3天静脉输入，其中有4例患者顺铂1天输注完毕。止吐方案：第1天，阿瑞匹坦125 mg，化疗输入前1 h口服，化疗前30 min输注托烷司琼5 mg，地塞米松10 mg。第2、3天，清晨口服阿瑞匹坦80 mg，化疗前输注托烷司琼、地塞米松。有效性评估从顺铂输注时起到第5天，即化疗药输注后0-120 h。在此期间，若患者出现Ⅲ度以上胃肠道反应，可给予解救的止吐药物。

### 1.2 观察目标

观察期可分为三个阶段：整体观察期：0 h-120 h（化疗后0 d-5 d）；急性呕吐观察期：0 h-24 h（化疗后1 d）；延迟性呕吐观察期：化疗后25 h-120 h（化疗后2 d-5 d）。输注化疗药物后，记录了患者胃肠道反应的时间、频度、分级、缓解方式。根据世界卫生组织（World Health Organization, WHO）分级标准将胃肠道反应分为0度-Ⅳ度，无、恶心、短暂呕吐、呕吐需治疗、难以控制的呕吐。Ⅲ度以上的呕吐需采取解救措施。本研究的观察目标包括：完全应答[没有呕吐，并且不需止吐的解救药物，完全缓解（complete response, CR）]；完全预防[没有恶心呕吐，完全预防（complete prevention, CP）]。

### 1.3 统计学方法

应用SPSS 21.0统计软件进行数据分析，率的比较采用*Pearson*卡方检验和*Fisher’s*确切概率法，以*P* < 0.05为差异有统计学意义。

## 2 结果

### 2.1 患者的一般特征

93例恶性肿瘤患者，年龄29岁-81岁，平均年龄为63岁，其中1例患者年龄超过80岁。患者具体情况见表 1。

**1:** 患者的一般特征（*n*=93） General characteristics of the patients (*n*=93)

Item	Patients
Total	93
Age (yr)	
< 65	58 (62.3%)
65-75	25 (26.9%)
> 75	10 (10.8%)
Gender	
Male	66 (71.0%)
Female	27 (29.0%)
ECOG PS score	
0	71 (76.3%)
1	22 (23.7%)
TNM stage	
Ⅳ	93 (100%)
Lung cancer	69 (74.2%)
Gastrointestinal tumor	24 (25.8%）
History of alcohol	
No drinking	48 (51.6%)
Drinking	45 (48.4%)

### 2.2 阿瑞匹坦治疗后患者的反应情况

整体观察期：达到CR的患者为86.02%（80/93），CP为46.22%（43/93）；急性呕吐观察期：CR为89.25%（83/93），CP为83.87%（78/93）；延迟性呕吐观察期：CR为87.1%（81/93），CP为45.16%（42/93）。见图 1。在延迟呕吐观察期，超过80%的患者无需处理，近半数病人达到完全预防。其中有4例患者顺铂1天输注完毕，有3例患者在整体观察期达到了完全预防，有1例患者只达到了完全反应。Ⅳ度呕吐反应3例，Ⅲ度呕吐反应7例，予以补救治疗后有8例可缓解。

**1:**
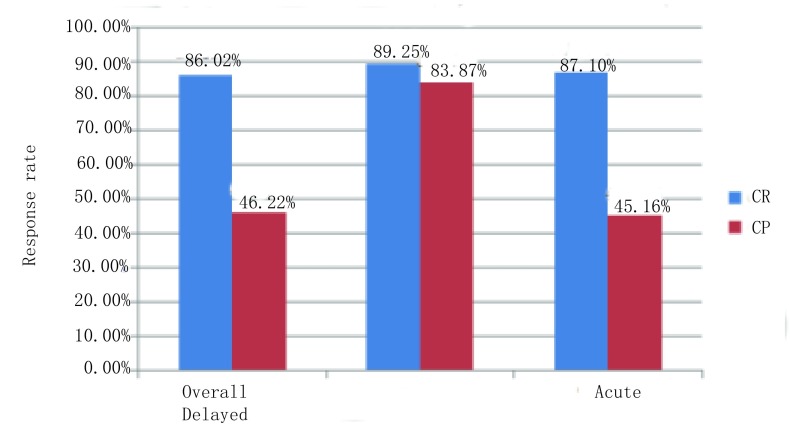
阿瑞匹坦治疗后患者的反应率 Patient response rate by aprepitant

### 2.3 各种因素对阿瑞匹坦止吐疗效的影响

我们统计了不同肿瘤、性别、年龄、体力状况的恶性肿瘤患者使用阿瑞匹坦止吐反应情况，阿瑞匹坦止吐效果与患者的恶性肿瘤类型无关（χ^2^=1.328, *P*=0.515）；止吐效果与性别无关（χ^2^=2.725, *P*=0.256）；阿瑞匹坦止吐效果与患者的体力状况无关（χ^2^=2.467, *P*=0.291）。但止吐效果与患者年龄分布关系具有显著性（χ^2^=12.854, *P*=0.008）（表 2）。

**2:** 各种影响因素与阿瑞匹坦止吐疗效的关系 The relationship between the effect factors and the efficacy of Aprepitant

Item	CR (0-120 h)	Invalid (0-120 h)	CP (0-120 h)	*χ*^2^	*P*
Tumor				1.328	0.515
Lung cancer	63 (61.2%)	6 (5.8%)	34 (33.0%)		
Gastrointestinal	20 (52.6%)	4 (10.5%)	14 (36.8%)		
Age (yr)				12.584	0.008
< 65	56 (65.9%)	2 (2.4%)	27 (31.8%)		
65-75	20 (52.6%)	5 (13.2%)	13 (34.2%)		
> 75	6 (46.2%)	4 (30.8%)	3 (23.1%)		
Gender				2.725	0.256
Male	61 (60.4%)	6 (5.9%)	34 (33.7%)		
Female	22 (61.1%)	5 (13.9%)	9 (25.0%)		
ECOG PS score				2.467	0.291
0	64 (62.7%)	7 (6.9%)	31 (30.4%)		
1	17 (53.1%)	5 (15.6%)	10 (31.2%)		

### 2.4 联合阿瑞匹坦止吐治疗的安全性

在使用阿瑞匹坦联合地塞米松、托烷司琼止吐的患者中未发现不可耐受的毒副作用，见表 3，90%以上患者无不适主诉。

**3:** 使用阿瑞匹坦止吐后相关不良事件发生情况 The adverse events occurred after the use of aprepitant

Adverse events	Number of patients	Ratio
Poor appetite	9	9.7%
Fatigue	10	10.8%
Constipation	6	6.5%
Diarrhea	1	1.1%
Headache	4	4.3%

## 3 讨论

在肿瘤的临床治疗中，化疗仍是不可或缺的治疗方法。铂类药物，特别是顺铂，是最常用的化疗药物之一，它属于高致吐的化疗药^[4]^，若不加处理，会引起90%以上患者发生CINV。CINV可以影响患者的生活质量，降低患者依从性，严重时可引起电解质紊乱、营养不良，部分患者因此而停止化疗，丧失治疗机会^[5]^。严重的CINV还会引起消化道出血、感染引发循环衰竭而导致死亡。

1981年，Baker采用10 mg地塞米松作为止吐药，其效果明显优于安慰剂。Gralla等研究表明，大剂量胃复安（1 mg/kg-3 mg/kg）有效抑制化疗引起的呕吐，并一度成为止吐的标准治疗方案。但对于高致吐化疗药，大剂量胃复安的有效率也只有30%-40%，并且有严重的毒副作用^[6]^。化疗后止吐仍然是一大难题。研究表明，与CINV有关的主要神经递质包括5-HT3、P物质和大麻素，多巴胺、乙酰胆碱、组胺也起到了一定的作用^[7]^。其中，5-HT3是引起急性期呕吐的主要神经递质。第一代5-HT3抑制剂主要用于急性期，对延迟期的止吐效果只有2.6%。P物质则属于激肽家族的调节多肽，主要结合NK受体，在急性期与延迟期呕吐中均发挥作用，主导延迟期的呕吐。它与NK-1的亲和力最大，并且两者的分布十分类似^[8]^。阿瑞匹坦作为NK-1阻滞剂，在2003年获得了美国食品药品监督局的批准，用于高度致吐化疗方案所致的恶心呕吐。其后，它在北美欧洲和南美分别进行了052及054的两项研究^[9, 10]^，结果显示，对于接受顺铂≥70 mg/m^2^的患者，阿瑞匹坦的治疗效果均优于对照组。在052研究中，整体观察期的CR是73%，CP是52%（*P* < 0.001）；在054研究中，整体观察期的CR是63%，CP是43%（*P* < 0.001）。分层分析显示，无论是在急性CINV，或延迟性CINV中，阿瑞匹坦均显示了优势（*P* < 0.001），并且没有增加毒性。在071、130研究中，在不同的肿瘤类型中，含阿瑞匹坦的止吐方案，对于使用中度致吐化疗药诱发的CINV也有效^[11-13]^。阿瑞匹坦与地塞米松、5-HT3抑制剂联合预防高、中度CINV，已成为指南推荐的方案^[14]^。

我们回顾分析了2014年-2016年使用含高度致吐药顺铂化疗方案的肿瘤患者中，阿瑞匹坦联合托烷司琼、地塞米松预防CINV效果。结果显示，达到CR的患者在整体观察期有86.02%（80/93），急性呕吐观察期和延迟性呕吐观察期分别为89.25%（83/93）、87.1%（81/93）；CP分别是46.22%（43/93）、83.87%（78/93）、45.16%（42/93）。这些数据与国外的报道类似。在延迟呕吐观察期，有超过80%的CINV患者无需处理，证实了阿瑞匹坦对于延迟性呕吐的预防效果明显。

CINV的发生率与诸多因素有关，有研究报道，除了化疗药物剂量与疗程的影响外，还与年龄，性别，饮酒史，体力状况等有关^[15-17]^。我们的研究结果显示，止吐效果与患者的肿瘤类型、性别及体力状态无关，但与年龄分布具有显著相关性，阿瑞匹坦对于低年龄组的患者止吐效果更佳。由于阿瑞匹坦的副作用较轻，而且预防性止吐效果明显，临床上极大地提高了患者治疗的依从性。在我们的临床实践中也发现，在因故中断阿瑞匹坦预防CINV的患者中，若再次使用阿瑞匹坦，仍然可以获得良好效果。

总之，阿瑞匹坦联合5-HT3受体阻断剂、地塞米松预防CINV显示出很高的有效性，尤其是显著降低了延迟期恶心呕吐的发生率，且副作用轻微，提高了患者对高致吐化疗药物的耐受性，为广大临床肿瘤医师提供了更有效的止吐选择。
